# Impact of Cadmium Contamination on Fertilizer Value and Associated Health Risks in Different Soil Types Following Anaerobic Digestate Application

**DOI:** 10.3390/toxics11121008

**Published:** 2023-12-10

**Authors:** Ghulam Mustafa Shah, Umer Farooq, Zunaira Shabbir, Jianbin Guo, Renjie Dong, Hafiz Faiq Bakhat, Muhammad Wakeel, Ayesha Siddique, Naeem Shahid

**Affiliations:** 1 Key Laboratory for Clean Renewable Energy Utilization Technology, Ministry of Agriculture, College of Engineering, China Agricultural University, Beijing 100083, China; 2 Department of Environmental Sciences, COMSATS University Islamabad, Vehari Campus, Vehari 61100, Pakistan; 3 Department of System-Ecotoxicology, Helmholtz Centre for Environmental Research—UFZ, Permoserstraße 15, 04318 Leipzig, Germany; 4 Department of Evolutionary Ecology and Environmental Toxicology, Goethe University Frankfurt, 60629 Frankfurt am Main, Germany

**Keywords:** Cd contamination, microbial biomass, biogas residues, nitrogen utilization, health risk assessment

## Abstract

Cadmium (Cd) contamination in the soil potentially hampers microbial biomass and adversely affects their services such as decomposition and mineralization of organic matter. It can reduce nitrogen (N) metabolism and consequently affect plant growth and physiology. Further, Cd accumulation in plants can pose health risks through vegetable consumption. Here, we investigated consequences of Cd contamination on fertilizer value and associated health risks following the application of biogas residues (BGR) to various soil types. Our results indicate that the application of BGR to all soil types significantly increased dry matter (DM) yield and N uptake. However, the Cd contamination negatively affected DM yield and N recovery from BGR in a dose-dependent manner. Organic N mineralization from BGR also decreased in Cd-contaminated soils. The highest DM yield and N recovery were recorded in sandy soil, whereas the lowest values were observed in clay soil. Cadmium was accumulated in spinach, and health risk index (HRI) associated with its dietary intake revealed that consuming spinach grown in Cd-contaminated soil, with or without BGR, is unsafe. Among the soil types, values of daily intake of metals (DIM) and HRI were lowest in clay soil and highest in sandy soil. However, the application of BGR curtailed HRI across all soil types. Notably, the application of BGR alone resulted in HRI values < 1, which are under the safe limit. We conclude that soil contamination with Cd reduces fertilizer value and entails implications for human health. However, the application of BGR to the soil can decrease Cd effects.

## 1. Introduction

Heavy metal contamination of soil has become a serious concern for the environment and human health. Among heavy metals, cadmium (Cd) is a highly toxic, carcinogenic and non-essential trace element found in soil. It has deleterious effects on soil organisms even at low concentrations [1]. According to the priority list of the Agency for Toxic Substances and Disease Registry (ATSDR), Cd is ranked seventh among toxic heavy metals [2].

In the environment, Cd pollution may arise from both natural and man-made activities [3,4]. Naturally, Cd is released from the lithosphere, sedimentary rocks, and soil through weathering processes [5]. Human-induced sources of Cd contamination include fuel emissions, manufacturing, wastewater irrigation, waste dumping, fertilizers and mineral mining [6]. Once Cd enters the soil, it can have adverse effects on soil microbes and may impact nutrient utilization from the soil and/or added organic materials [7]. The accumulation of cadmium (Cd) in soil can disrupt microbe-mediated processes such as decomposition and mineralization, which ultimately alters the ecological balance [8].

Different factors including soil type and environmental variables are very relevant for the bioavailability of Cd to soil microbes, thereby alter impacts on microbial activities [9]. Numerous studies have shown that soil types can affect Cd bioavailability through adsorption, cation exchange, precipitation with chemical agents and complexation with organic and inorganic ligands [10,11]. Additionally, clay content imparts a significant role in sequestration of Cd ions, and thereby reducing toxicity to soil microbes.

Cadmium is highly mobile within the soil–plant system and, exert toxic effects on plants, and may induce human health risks through food chain [12]. As a non-essential heavy metal, Cd does not play any biological role in plant metabolic functions. However, after uptake by plants, it accumulates in roots, shoots, grains and leaves, disrupts N metabolism and the physiological functioning and leads to growth inhibition and imbalance of micronutrients. Consuming Cd-contaminated vegetable can have serious health implications [13]. Approximately 70–80% of Cd intake by humans comes from the consumption of vegetables grown in Cd-contaminated soils [14]. In the human body, Cd can adversely affect the lungs and liver and may even contribute to the development of multi-organ cancer [15]. Considering health and ecological consequences of Cd, it is highly imperative to remediate Cd-contaminated soils.

Different scientific approaches including physically excavating contaminated soil, landfilling and chemical-assisted phyto-extraction of pollutants have been applied to remediate Cd-contaminated soils. However, these methods are costly and environmentally disruptive [16,17]. Since Cd is non-degradable in nature, it can be stabilized in soil by adding organic amendments, which are efficient in reducing its availability by adsorption, binding and co-precipitation mechanisms [18]. In recent decades, Cd availability in soil has been successfully reduced by applying compost, biochar and other organic sources rich in organic matter [19]. However, they focused on evaluating Cd’s fate in soil through incubation studies, with less emphasis on evaluating its impacts on microbial activities and fertilizer value of organic materials.

In Pakistan, the production of BGR has increased due to subsidized installation of biogas plants. The BGR is produced after anaerobic digestion of farmyard manure or organic material (including crop residues) in biogas plants. This material is rich in organic matter and mainly used as a nutrient source for vegetables and crops. The use of organic material can decrease the bioavailability of Cd in soil, lower the uptake by plants and thereby reduce associated health implications. We hypothesized that Cd may affect soil microbes and their activities such as mineralization and decomposition of added BGR. Furthermore, soil texture and BGR may alter Cd bioavailability for both plants and soil microbes, which may impact (i) microbe-mediated soil processes such as decomposition and mineralization and (ii) Cd uptake by plants and, consequently, its associated health risks. Accordingly, we aimed at investigating the effects of Cd on plant bioaccumulation, associated human health risks and fertilizer value after applying BGR to different soil types.

## 2. Materials and Methods

### 2.1. Experimental Setup

To investigate the impact of Cd on N mineralization and recovery from BGR and health risks associated with Cd accumulation in spinach, we conducted outdoor pot experiments. The BGR was obtained from a nearby biogas plant, and characteristics are given in Table 1.

We collected three distinct soil types, viz. sandy soil, clay soil and sandy clay soil, from identified locations in the surrounding areas. Their physico-chemical characteristics are provided in Table 2. To ensure consistency, all three soil types were homogenized and sieved through a 4 mm mesh screen. Each pot was filled with 7 kg of soil, providing a total surface area of 0.035 m^2^. For metal exposure, we introduced four different Cd concentrations (0, 20, 40 and 60 ppm) by spiking irrigation water into pots containing various soil types. According to local agricultural recommendations, BGR was incorporated into the top 15 cm soil profile at a rate of 55 kg N per acre after two weeks.

Approximately ten seeds of spinach were sown in each pot four days after the application of treatments. For the data collection and analysis, only five healthy seedlings were maintained in each pot. Control groups of each soil type (with or without Cd concentration) were included in the experiment where no BGR was applied. All treatments were arranged in completely randomized design with factorial arrangements, and each was replicated three times. Throughout the experiment, the moisture content in pots was maintained at 60%.

### 2.2. Plant Harvesting and Soil and Plant Analysis

The spinach plants were harvested at different time points: after 30, 60 and 90 days. During each harvest, plants were clipped 1 cm above the soil surface. The collected shoot samples were air dried followed by oven drying for 48 h at 70 °C. Roots were harvested at the end of the experiment, following the procedure described by Shah, Tufail [20]. The dried shoot and root samples were ground to pass through a 1 mm sieve. Representative samples were analyzed for their total nitrogen (N) using a Kjeldahal apparatus, and Cd using the Atomic Absorption Spectrometer (PerkinElmer^®^- PinAAcle™ 900F, AAS).

For BGR and soil, selective chemical analyses were conducted to assess various properties, including pH, electric conductivity (EC), total carbon (C), total nitrogen (N), mineral N, Cd content, organic matter, lignin, cellulose and hemicelluloses, performed following the methods described earlier [21].

### 2.3. Fertilizer Value

#### Nitrogen Recovery and Mineralization

The total nitrogen recovery (TNR) by plants from soil treated with anaerobic digestate was calculated by using Equation (1):

(1) $$TNR\%=\frac{{(TNU}_{Treatement}-{TNU}_{Control})}{{TN}_{Applied}}\times 100$$

where TNU_Treatment_ is the plant uptake of nitrogen from soil applied with anaerobic digestate, TN_Control_ represents amount of total N ending up in plants from control and TN_Applied_ is the quantity of total nitrogen applied to soil via digestate application.

To calculate the organic N mineralized from BGR, we used total N uptake by plants together with the residual soil mineral N content (Equation (2), [22]).

(2) $$N_{Min}=\left({TNU}_{Manure}+\mathrm{Residual}\;\mathrm{soil}\;\mathrm{mineral}\;N\right)-\mathrm{mineral}\;N\;\mathrm{applied}$$

### 2.4. Associated Health Risks Measurement

#### 2.4.1. Daily Intake of Metals (DIM)

To estimate daily spinach consumption (kg day^−1^ per person), a survey was conducted among residents of Vehari city, specifically targeting individuals aged 25–35, both male and female. The mean Cd concentrations in spinach (mg kg^−1^) were determined in shoot samples using an atomic absorption spectrometer. To assess the daily intake of Cd (mg kg^−1^) through dietary consumption of spinach, the following equation was employed [23].

(3) DIM=Daily spinach Consumption×Mean spinach Cd Concentration

#### 2.4.2. Health Risk Index (HRI)

The human health risk index (HRI) from Cd ingestion through contaminated spinach was determined using the relationship below [24].

(4) $$HRI=\frac{\left(DIM\right)\times (C\;Metal)}{RD\times Bo}$$

The concentration of Cd in spinach was expressed as C Metal. RD represents the globally recognized oral reference dose for Cd, which is equivalent to 0.001 mg kg^−1^. B_0_ denotes the average human body weight of 70 kg [25].

### 2.5. Statistical Analysis

The data were statistically evaluated in STATISTIX 8.1 using an analysis of variance (ANOVA). When the main effect was statistically significant, the least significant difference (LSD) test was employed to compare treatment means at the 5% probability level.

## 3. Result

### 3.1. Fertilizer Value

#### 3.1.1. Total Dry Matter Yield of Spinach

We observed that, in comparison to the unfertilized control, the application of BGR significantly increased the dry matter yield in sandy soil, clay soil and sandy clay soil by 84% (611 vs. 1100 g m^−2^), 80% (393 vs. 724 g m^−2^) and 81% (500 vs. 905 g m^−2^), respectively. Among the soil types, the highest total dry matter yield of spinach was observed in sandy soil and the least in clay soil. However, the addition of Cd significantly reduced the total dry matter yield of spinach (Table 3), showing a dose-dependent effect. For instance, 20 ppm of Cd decreased dry matter yield of spinach by 6% in sandy soil, 3% in clay soil and 5% in sandy clay soil. On the other hand, 40 ppm of Cd reduced the dry matter yield by 12% in sandy soil and 9% in clay and sandy clay soils. In the case of the highest Cd concentration (60 ppm), the reduction in dry matter yield was 18% in sandy soil, 17% in clay soil and 14% in sandy clay soil. Although reduction was stronger in sandy soil, the dry matter yield of spinach was still higher as compared to other soil types (clay and sandy clay soils). When Cd was applied in BGR-amended soils, the total dry matter yield of spinach was also highest in sandy soil. Thus, the order of soil types in terms of dry matter yield followed this pattern: sandy soil > sandy clay > clay soil.

#### 3.1.2. Apparent N Recovery in Spinach Plant

**Figure 1:**
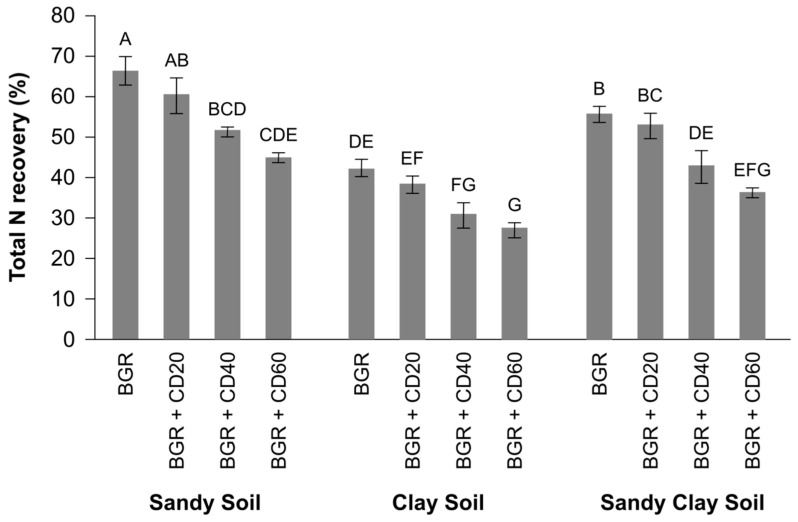
Total nitrogen recovery by spinach from biogas residues was assessed in sandy, clay and sandy clay soils contaminated with different levels of cadmium. Error bars (±) represent standard error of the mean (*n* = 3), whereas bars labeled with capital letters indicate significant differences at a 5% probability level.

Total plant N uptake was used to calculate apparent nitrogen recovery (ANR) by spinach plants from BGR with and without addition of Cd. When BGR was applied solely in sandy, clay and sandy clay soils, the ANR was 66%, 42% and 58%, respectively (Figure 1). Within each soil type, the ANR fraction from BGR decreased with increasing Cd contamination. For instance, in clay soil, the addition of Cd reduced the ANR value by 10% (42% vs. 38% of the N applied) at the lowest concentration (Cd 20 ppm), 27% (42% vs. 31%) at a medium concentration (Cd 40 ppm) and 42% at (42% vs. 25%) at highest (60 ppm) Cd concentrations. Among different soil types, the highest ANR recovery from BGR, with or without Cd, was observed in sandy soil, whereas the lowest was observed in clay soil (Figure 1).

#### 3.1.3. N Mineralization

Our results indicate that approximately 31%, 26% and 8% of the applied organic N through BGR were mineralized in sandy, sandy clay and clay soils, respectively (Table 4). However, in soils contaminated with 60 ppm of Cd, these fractions were reduced to 10%, 1% and −10%, respectively. The net soil N immobilization from BGR was observed in clay soil.

### 3.2. Human Health Risks

The results showed a significant increase in shoot Cd concentration with higher Cd levels in soil. However, application of BGR to the soil decreased shoot Cd concentration, suggesting a decrease in Cd bioavailability due to the presence of the organic material. A similar trend was recorded for DIM. On average, the reduction in shoot Cd was 14% in sandy soil, 11% in clay soil and 22% in sandy clay soil. The type of soil had an impact on DIM, with the lowest average values found in sandy soil and the highest in sandy clay soil. Further, the application of BGR to the soil decreased DIM values in sandy soil, clay soil and sandy clay soil by 14%, 6% and 23%, respectively.

The presence of Cd had a substantial impact on the HRI values (Table 5). The results revealed that the application of Cd increased the HRI values, showing dose-dependent effects regardless of BGR application. The highest concentration of Cd showed highest HRI values in all soil types: sandy soil (10.02), sandy clay soil (6.85) and clay soil (6.38). However, the application of BGR significantly reduced HRI values in all soil types, with reductions of up to 50%.

## 4. Discussion

### 4.1. Fertilizer Value (DM Yield, N Recovery and N-Mineralization)

Our results indicate that applying BGR increased spinach DM yield compared to the unfertilized control. This improvement might be attributed to enhanced nutrient availability, especially nitrogen, resulting from BGR mineralization. BGR also enhances water retention, improves soil structure for better aeration and provides a nutrient source for microbial growth. Biogas residues, the end product of anaerobic fermentation of organic materials in biogas plants, release easily degradable organic N fractions, facilitating rapid nutrient release when applied to the soil [26,27,28]. Studies have shown that in clay soil, BGR application immobilizes a portion of ammonium-N (NH_4_^+^-N) through microbial action, likely due to the negatively charged clay surface. Mineralization and immobilization of organic N from applied waste, such as residues, are influenced by factors such as soil characteristics, microbial activities, and environmental conditions and soil texture [29].

In our study, we observed that the DM yield and ANR from BGR were affected by soil types, with the highest values in sandy soil and the lowest in clay soil (Figure 1). These findings are consistent with Shah, Rashid [26], who reported decreased N recovery and DM values when poultry manure, farmyard manure and slurry were applied to clay soil compared to sandy or peat soils. This could be attributed to higher clay content, which entraps organic material and decreases N mineralization. Earlier studies have also showed that soil clay content is negatively associated with net N mineralization rate of applied organic manures [29]. Reasons for this process include (i) NH_4_^+^-N fixation into clay minerals’ interlayer spaces [30], (ii) entrapment of organic N compounds in soil aggregates, making them inaccessible to soil microbes, and (iii) the physical protection of microbial biomass in the soil structure [31]. Thus, a significant portion of the high applied N through BGR becomes sequestered in the soil and is protected from decomposition by decomposers such as nematodes and micro-arthropods. In our study, these factors might be the reason for lower N mineralization rate and net immobilization in clay soil (Table 4). In contrast, sandy soil showed greater N recovery and mineralization. It is suggested that sandy soil provides better aeration and less protection for organic material, leading to relatively greater N mineralization. Our results align with Shah, Rashid [26], who also observed net immobilization when farmyard manure (FYM) and cattle slurry were applied to clay soils and concluded that organic waste such as manures applied to sandy soil results in greater N recovery and mineralization compared to clay soils.

Regardless of the soil types, Cd contamination in soil significantly reduced DM yield and N recovery from BGR as compared to its sole application. This reduction can be attributed to reduced microbial biomass, compromised biochemical processes and less decomposition of organic matter [32]. Further, Cd in the plant system has been associated with the overproduction of reactive oxygen species (ROS), leading to membrane damage and elevated malondialdehyde (MDA) contents in spinach [33]. Cd can denature the microbial cell and substitutes Zn in metabolic processes. The replacement of micro-nutrients by Cd is one of the toxic actions, which results in oxidative stress and triggers the DNA damage and apoptosis [34]. Stoimenov, Klinger [35] also reported that heavy metals decrease microbial activity and slow down the decomposition process. However, application of organic waste amendments is a suitable option for Cd-contaminated soils.

### 4.2. Health Risk Assessment

Our results revealed that Cd bioaccumulation in plants and DIM and HRI values increased with increasing Cd in soil. Further, the highest DIM and HRI values were found in sandy soil, whereas the lowest in clay soil. This indicates a significant influence of clay content on the bioavailability of Cd. These results are consistent with earlier investigations that have shown that clay fractions in soil can adsorb heavy metal ions, including Cd ions, through specific adsorption and ion exchange processes [36]. In terms of adsorption, metals generally show significant affinity for the clay fraction, with the ranking typically following clay > silt > sand [37].

Cadmium contamination (*p* < 0.05) and soil types had a substantial impact on HRI values (Table 5). In our study, HRI values of spinach were consistently ≥1 in almost all Cd treatments, both with and without the addition of BGR, indicating it is unsafe to consume spinach grown in Cd-contaminated soils. However, the addition of BGR decreased the HRI values, regardless of the soil types. This reduction could be attributed to precipitation, complexation and co-precipitation of Cd by BGR. Previous research has shown that the amount of organic matter in soil significantly influences absorption and translocation of heavy metals in soil and their uptake in plants. Cd tends to be adsorbed onto organic materials, leading to stable forms and accumulation in the organic horizons of soil [38]. Our results align with Bolan, Kunhikrishnan [39], who observed that the application of organic amendments reduced heavy metals in the soil solution by immobilizing them in the solid phase. Several investigations have reported significant reduction in Cd accumulation in vegetables and crops when they applied organic amendments [40,41,42].

Application of organic waste increases soil pH, which may decrease the availability of Cd [43]. Organic waste surfaces possess exchange sites that significantly influence the retention and limited availability of elements [44]. Vickers [45] also reported the retention of Cd on the surface of organic waste and demonstrated that this sorption is not an instantaneously reversible process. The high sorption capacity of Cd on organic waste surfaces may be attributed to factors such as electrostatic interactions between negatively charged organic matter surfaces and Cd cations, ionic exchange between Cd and the ionizable portions of organic waste surfaces and sorptive interactions [46]. Further, it is suggested that the metal speciation and bioavailability in soil are closely linked to soil pH, a key factor affecting the mobility of labile elements such as Zn, Ni and Cd [47]. Decreasing the soil pH increases the mobility of these elements. To alleviate metal pollution, organic wastes such as lime and manure are frequently applied, which can raise soil pH and decrease the metal toxicity [48].

Contrary to our findings, Saleem, Riaz [49] reported elevated Cd concentrations in spinach when compost was added to different treatments. However, the compost was already contaminated with heavy metals. This might be contributing to the higher bioaccumulation of Cd. The composition of materials used in organic waste preparation play a significant role in transfer of trace metals into the food chain through organic waste additions [50]. Municipal or city waste typically contains higher trace metal content, while organic manure derived from agricultural residues, such as crop residues, tends to have lower trace metal content [51].

In humans, consumption of contaminated foods is one of the main routs of Cd exposure [52]. However, the application of organic wastes to polluted soils can significantly reduce Cd bioaccumulation in plants. As a result, daily consumption and HRI can be mitigated. Our results elucidate that the application of BGR can be helpful in decreasing bioaccumulation of metals in vegetables and thereby reducing the associated human health risks.

## 5. Conclusions

This study found that Cd contamination of soil negatively affects BGR mineralization, plant yield and N recovery. Higher Cd contamination leads to increased health risk indices. However, adding BGR to Cd-contaminated soil improves yield, reduces Cd bioavailability to plants and lowers Cd content, DIM and HRI. Across soil types, the order for these parameters is sandy soil > sandy clay soil > clay soil. We conclude that the application of BGR on Cd-contaminated soil restricts Cd uptake and enhances microbial mediated N mineralization and plant yield.

## Figures and Tables

**Table 1:** Mean (*n* #3) characteristics of biogas residues (BGR) used in this study. Values in the parenthesis represent the ± standard error of the mean.

Parameters	Units	BGR
Dry matter	%	55.14 (±2.16)
Organic matter	%	30.54 (±2.50)
N_total_	%	3.56 (±0.04)
N_min_	%	0.98 (±0.06)
Total C	%	46.54 (±3.92)
C:N ratio	-	13.05
Cd	mg kg^−1^ DM	2.23 (±0.447)
pHH_2_O 1:10	-	7.45 (±0.18)
EC (1:5)	dS m^−1^	3.02 (±0.29)
Lignin	% DM	28 (±1.60)
Cellulose	% DM	26 (±0.97)
Hemicellulose	% DM	20 (±1.23)

**Table 2:** Mean (*n* #3) characteristics of sandy soil, clay soil and sandy clay soil used in this study. Values in the parenthesis represent the ± standard error of the mean.

Parameters	Units	Sandy Soil	Clay Soil	Sandy Clay Soil
Dry matter	%	95.96 (±9.82)	42.54 (±3.30)	55.14 (±2.16)
Organic matter	%	16.92 (±1.33)	27.60 (±5.72)	30.54 (±2.50)
N_total_	%	01.23 (±0.10)	3.75 (±0.02)	3.56 (±0.04)
Total C	%	19.80 (±0.12)	51.36 (±5.61)	46.54 (±3.92)
C:N ratio	-	16.09	13.69	13.05
Cd content	mg kg^−1^ DM	2.4 (±0.447)	2.23 (±0.447)	2.5 (±0.447)
pH_H2O_ 1:10	-	7.1 (±0.90)	7.3 (±1.0)	7.3 (±0.18)
EC (1:5)	dS m^−1^	243.4 (±0.07)	306.7(±0.76)	286.7 (±0.29)
Sand	%	60 (±6.33)	40 (±2.16)	52 (±3.52)
Silt	%	30 (±5.72)	30 (±2.50)	28 (±5.72)
Clay	%	10 (±0.02)	30 (±9.82)	20 (±1.33)

**Table 3:** Dry matter (DM) yield and nitrogen (N) uptake in spinach. Values followed by different letters within a column are significantly different from each other.

Treatments	Total Dry Matter (g m^−2^)			N Uptake (g m^−2^)			N Use Efficiency		
	Sandy Soil	Clay Soil	Sandy Clay Soil	Sandy Soil	Clay Soil	Sandy Clay Soil	Sandy Soil	Clay Soil	Sandy Clay Soil
Control	611.43 c	393.33 b	500.48 c	6.23 c	4.00 d	5.18 c	98.2	98.33	96.56
Cd20	595.24 c	390.71 b	494.29 c	6.24 c	3.99 d	5.14 c	95.39	98.01	96.24
Cd40	585.71 c	387.33 b	486.67 c	6.23 c	4.02 d	5.18 c	94.02	96.37	93.98
Cd60	578.57 c	379.05 b	484.95 c	6.31 c	3.90 d	5.20 c	91.73	97.2	93.34
BGR	1100 a	723.81 a	904.76 a	12.85 a	8.23 a	10.74 a	85.59	87.97	84.23
Cd20 + BGR	1038.1 ab	704.76 a	857.14 ab	12.26 a	7.80 ab	10.42 a	84.69	90.31	82.25
Cd40 + BGR	971.43 ab	657.14 a	819.05 ab	11.36 ab	7.09 bc	9.44 ab	85.51	99.47	86.79
Cd60 + BGR	904.76 b	600.00 a	780.95 b	10.79 b	6.60 c	8.83 b	83.89	106.7	88.47

**Table 4:** N balance based on N applied, N uptake and final mineral N in soil. Values followed by different letters within a column are significantly different from each other.

	N Applied (g m^−2^)			N Uptake by Spinach (g m^−2^)		Residual N_min_ (g m^−2^)		Net N_Organic_ Mineralized	
	Total	Organic	Mineral	Total	From BGR	Total	From BGR	A	B
Sandy Soil									
Control	0.00	0.00	0.00	6.23	–	0.67	–	–	–
Cd_60_	0.00	0.00	0.00	6.23	–	0.58			
BGR	13.50	9.32	4.19	12.85	6.63	1.09	0.42	2.86	30.75 a
BGR + Cd_60_	13.50	9.32	4.19	10.79	4.48	1.16	0.89	0.93	10.04 c
Sandy Clay Soil									
Control	0.00	0.00	0.00	5.18	–	0.45	–	–	–
Cd_60_	0.00	0.00	0.00	5.01	–	0.42	–	–	–
BGR	13.50	9.32	4.19	10.74	5.56	0.91	0.46	1.82	19.74 b
BGR + Cd_60_	13.50	9.32	4.19	8.83	3.82	0.88	0.46	0.10	1.04 d
Clay Soil									
Control	0.00	0.00	0.00	4.00		0.27	–	–	–
Cd_60_	0.00	0.00	0.00	3.90		0.30	–	–	–
BGR	13.50	9.32	4.19	8.22	4.23	0.97	0.70	0.75	8.00 cd
BGR + Cd_60_	13.50	9.32	4.19	6.60	2.48	0.83	0.36	0.95	–10.19 e

**Table 5:** Health risk assessment of Cd metal via dietary intake of spinach applied with BGR. Values followed by different letters within a column in a single vegetable are significantly (*p* < 0.05) different from each other.

Treatments	C Metal Cd (mg kg^−1^)			DIM (mg day^−1^)			HRI		
	Sandy Soil	Clay Soil	Sandy Clay Soil	Sandy Soil	Clay Soil	Sandy Clay Soil	Sandy Soil	Clay Soil	Sandy Clay Soil
Control	4.3 e	6.3 e	7.5 d	0.0031 e	0.0045 c	0.0063 d	0.19 c	0.41 c	0.75 e
Cd20	9.8 de	11.8 bcd	17.7 b	0.0070 de	0.0085 bc	0.0150 b	1.02 c	1.66 bc	3.97 bc
Cd40	18.7 bc	17.2 b	20.5 ab	0.0134 bc	0.0124 abc	0.0174 ab	3.6 c	3.09 abc	5.12 ab
Cd60	30.3 a	24.4 a	23.6 a	0.0218 ab	0.01766 a	0.0200 a	10.0 a	6.38 a	6.85 a
BGR	7.1 e	7.8 de	7.3 d	0.0051 e	0.00567 bc	0.0062 d	0.51 c	0.68 c	0.69 e
Cd20 + BGR	8.8 e	9.7 cde	12.2 c	0.0063 e	0.0070 bc	0.0103 c	0.81 c	0.97 c	1.83 de
Cd40 + BGR	15.6 cd	14.2 bc	16.5 b	0.0112 cd	0.0102 abc	0.0140 b	2.61 bc	2.10 bc	3.35 cd
Cd60 + BGR	25 ab	24.8 a	19.3 b	0.0180 ab	0.0208 ab	0.0164 b	6.49 ab	4.9 ab	4.53 bc

## Data Availability

Data are contained within the article.
